# Perivascular mesenchymal stem cells in the adult human brain: a future target for neuroregeneration?

**DOI:** 10.1186/2001-1326-1-30

**Published:** 2012-11-23

**Authors:** Ilknur Özen, Jordi Boix, Gesine Paul

**Affiliations:** 1 Department of Clinical Sciences, Translational Neurology, Lund University, Wallenberg Neuroscience Center, Lund 22 184, Sweden; 2 Department of Neurology, Scania University Hospital, Lund 22 185, Sweden

**Keywords:** Pericytes, Mesenchymal stem cells, Perivascular niche, Neuroregeneration

## Abstract

Perivascular adult stem cells have been isolated from several tissues, including the adult human brain. They have unique signatures resembling both pericytes and mesenchymal stem cells. Understanding the nature of these cells in their specific vascular niches is important to determine their clinical potential as a new adult stem cell source. Indeed, they have promising features *in vitro* in terms of multipotency, immunomodulation and secretion of growth factors and cytokines. However, their *in vivo* function is less known as yet. Recent emerging data show a crucial role of perivascular mesenchymal stem cells in tissue homeostasis and repair. Furthermore, these cells may play an important role in adult stem cell niche regulation and in neurodegeneration. Here we review the recent literature on perivascular mesenchymal stem cells, discuss their different *in vitro* functions and highlight especially the specific properties of brain-derived perivascular mesenchymal stem cells. We summarize current evidence that suggests an important *in vivo* function of these cells in terms of their regenerative potential that may indicate a new target cell for endogenous tissue regeneration and repair.

## Review

### Adult stem cells

Adult stem cells (ASCs) are found in almost all organs of the postnatal human body. They reside in the perivascular niche, a specific microenvironment that allows ASCs to retain their multi-lineage potential and self-renewal capacity [1,2]. The perivascular niche consists of ASCs, neighbouring cells and extracellular matrix [1,3,4].

Adult stem cells are a source for organ-specific cell replacement either during the normal cell turnover or under pathological conditions [5,6]. These stem cells often remain dormant until they are activated by the body’s need to maintain tissues, or in response to disease or tissue injury. Some ASC types, such as hematopoietic stem cells (HSCs) or enteric stem cells, have a high proliferation rate, whereas in other organs, ASCs only divide under certain conditions, stimulated by injury for example.

In contrast to embryonic stem cells, the differentiation potential of ASCs is regarded as more restricted, usually to the cells of the tissue in which they reside. This suggests that the differentiation of an ASC into a specialized cell might be dependent on the surrounding tissue. However, this classical paradigm of tissue-specific differentiation capacity has been challenged by observations of a different degree of plasticity in some adult tissues that has resulted in differentiation beyond tissue boundaries [5].

## Mesenchymal stem cells

One ASC type that has specifically attracted attention during the past years are mesenchymal stem cells (MSCs) [7-12]. Friedenstein and co-workers [13] were the first to describe MSCs, originally termed mesenchymal stromal cells, as a rare population of plastic-adherent cells that could be isolated from the bone marrow but was different from HSCs [13]. Mesenchymal stem cells are isolated by adherence to plastic, possess a high proliferative potential and are characterized by the expression of a panel of surface markers [14] and their capacity to differentiate along mesodermal lineages such as osteoblasts, chondrocytes and adipocytes [15]. They have gained interest because they are not only multipotent, they also support hematopoiesis [16-18], are immunomodulatory [19-23] and have an intriguing pro-regenerative capacity due to the secretion of different growth factors and mitogens [12,23].

## Mesenchymal stem cells reside in the perivascular niche

Interestingly, sources for MSCs are not restricted to the bone marrow. Indeed, MSCs have been isolated from several tissues in different species [7,10] but also from different human tissues and organs [24-26] including bone marrow [27], dental pulp [27,28], adipose tissue [29], umbilical cord Wharton’s jelly [30], placenta [31] and recently also from the adult human brain [32]. Importantly, these MSCs are located in the perivascular niche and exhibit similarities to pericytes in terms of phenotype, gene expression and differentiation capacity [25,26,32].

Evidence, that MSCs and pericytes are biologically related had remained indirect for a long time, but a more systematic analysis of their association has only recently been made [25,26,33,34]. Now it has been shown that MSCs may reside in the perivascular compartment and have characteristics identical to a subclass of pericytes [10,24-26,32,34]. However, pericytes around capillaries are suggested not to be the only ancestors of MSC’s [33]. Adventitial cells that reside around larger vessels also natively express MSC surface markers [35,36].

## Pericytes

Pericytes are a heterogeneous cell population in the vascular niche [37], that line the abluminal side of endothelial cells in the perivascular space and are embedded within a shared basement membrane [38,39]. They span the entire microvasculature. The phenotypic identification of pericytes is rather difficult due to the lack of one specific pericyte marker. Therefore, besides their location, a panel of different markers is usually used to identify pericytes [38,40-42].

This diversity in pericyte marker expression may be due to differences in tissue location, vessel size or embryonic origin. It is generally proposed that pericytes are either mesodermal or neural crest-derived [43,44], depending on their location in any given organ. In addition to their multiple embryonic origins, pericytes may develop from several adult cell types [38,42,45]. In the resting stage, pericytes are quiescent slow-cycling cells [46]. Once isolated from different tissues, pericytes have the capacity to proliferate and differentiate into different cell types *in vitro*.

## Perivascular mesenchymal stem cells - a novel stem cell in the human brain

For many decades, the adult brain, in contrast to other tissues, was thought to not be capable of regeneration. However, it is now widely accepted that the adult human brain contains neural progenitors [47-53]. In the brain, adult neural stem cells are also found in specialized vascular niches, mainly in the neurogenic zones, the subventricular zone and the subgranular zone of the dentate gyrus [54-57]. In these vascular niches, the neural stem cells contact the vasculature at the sites that lack astrocyte endfeet and pericyte coverage [58].

Neural progenitor cells could also be derived from a variety of adult brain regions other than the known neurogenic zones [49,52,59,60]. Human adult progenitor cells isolated from non-neurogenic regions multiply *in vitro* and give rise to cells with the characteristics of neurons, astrocytes, and oligodendrocytes [59,61,62].

Analyzing human brain tissue from biopsies and temporal lobectomies, we have identified a novel adult stem cell with mesenchymal characteristics located around small blood vessels in the human brain that is different from the previously described neural stem and/or progenitor cells [32]. These perivascular cells expressing mesenchymal (CD105, CD13) and pericyte markers (PDGFR-β) are mainly located at vascular branching points. Some of the pericytes co-expressing MSC markers are proliferating cells. Isolated cells were further purified by fluorescence-activated cell sorting (FACS), gating them positively for CD105, CD13, and negatively for the hematopoietic marker CD45 and the endothelial marker CD31. The expanded purified cells exhibited a marker signature for both MSCs and pericytes *in vitro* (CD73, CD90, CD13, CD106, CD49d, PDGFR-β, RGS5, α-SMA, NG2). Cells were negative for hematopoietic, endothelial, and glial markers. Most importantly, the isolated cells did not express any of the neural progenitor markers that are typical for adult neural stem cells (CD133, SOX1, NGN2, PAX6 and Musashi) (Table 1).

**Table 1:** Marker expression of perivascular MSC isolated from the adult human brain

**POSITIVE EXPRESSION**	**NO EXPRESSION**	
**Pericyte Markers**	**Neural/ glial progenitor markers**	
PDGFR-β	PAX6	CD133
RGS5	A2B5	SOX1
α-SMA	S100b	Musashi
NG2	GLAST	Neurogenin2
Nestin	GFAP	Tuj1
Kir6.1	O4	Doublecortin
**Mesenchymal Markers**	**Endothelial markers**	
CD105	CD31	
CD13	CD34	
CD73	**Hematopoietic markers**	
CD90	CD45	
CD166	CD56	
CD49d	**Macrophage/microglia markers**	
CD29	CD14	
	CD11b	

**Figure 1:**
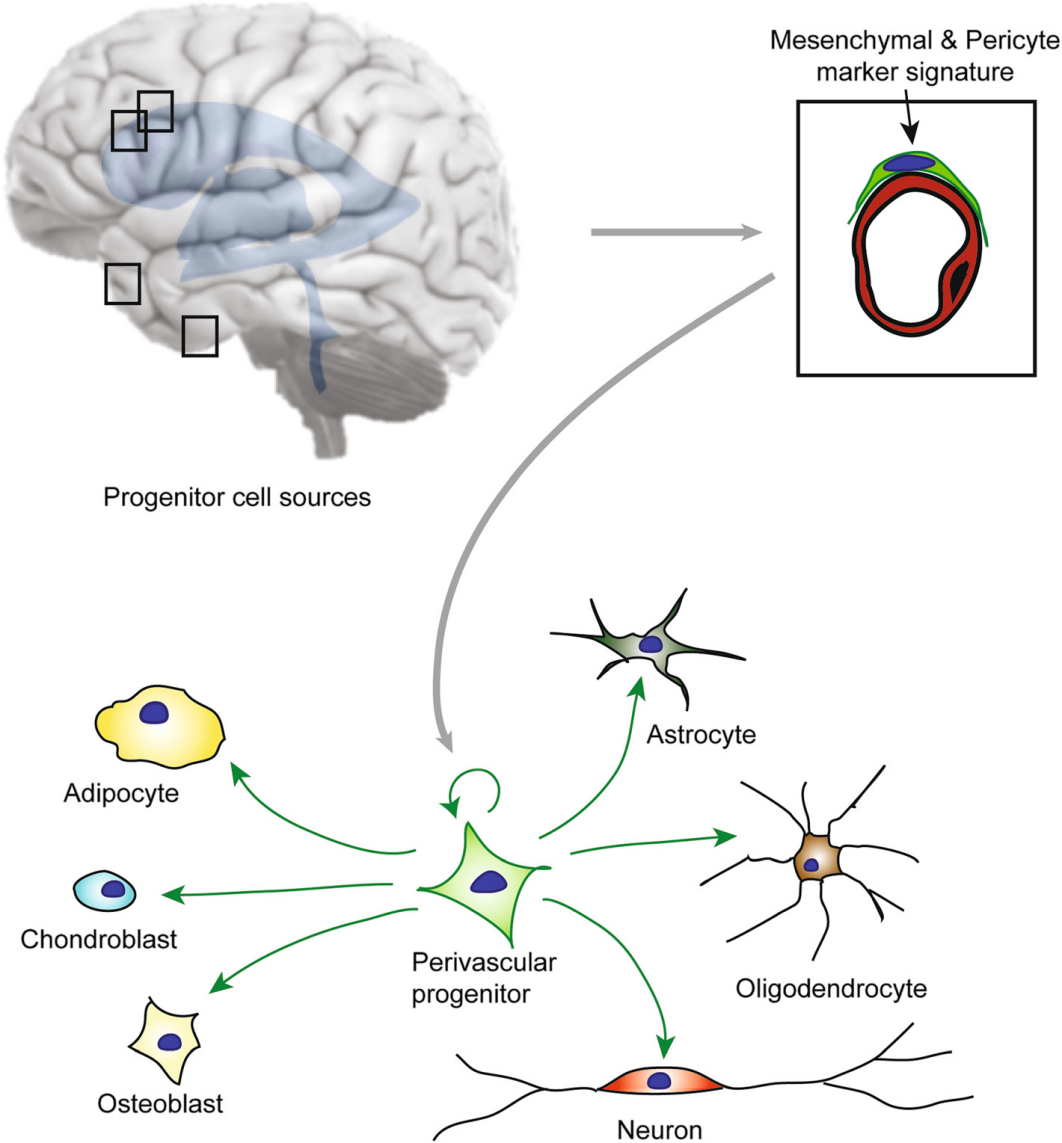
**The adult human brain vascular niche contains a novel progenitor with multi-lineage capacity that appears to represent both MSCs and pericytes.** These progenitor cells give rise to both neuronal lineage (astrocyte, oligodendrocytes, and neurons) and mesodermal lineage (adipocytes, chondroblasts, and osteoblasts) at the clonal level.

Isolated perivascular MSCs from the adult human brain undergo self-renewal *in vitro* and give rise to single-cell-derived clones that are indistinguishable from polyclonal perivascular MSCs in terms of adherence, morphology, proliferation, and surface antigen expression. Surprisingly, the capacity of these brain-derived perivascular MSCs was far superior to our initial expectations (Figure 1). Single-cell-derived clones gave rise to adipocytes, chondroblasts and osteoblast when exposed to the appropriate inductive signals, a feature that had been described for both MSC [15,25] and pericytes [25,63-66].

Most interestingly, when isolated perivascular MSCs were exposed to glial induction medium, the cells differentiated into oligodendrocytes or astrocytes, pericyte-specific antigens were downregulated and cells expressed glial fibrillary acidic protein (GFAP).

Furthermore, upon neuronal induction, the same perivascular MSC clones downregulated mRNA for pericyte markers (α-SMA, Nestin, RGS5, NG2 and PDGFR-β) and upregulated mRNA for neuronal transcription factors (NeuroD1, Pax6, Tbr1, Tbr2) and neuronal markers (DCX, Tuj1) and consistently expressed neuron-specific proteins (DCX; HuC/D, Map2, Tuj1, NSE). A proportion of neurons expressed the synaptic marker PSD95 and GABA A-receptor, indicating a more mature neuronal phenotype. Cells exhibited typical electrophysiological features of immature neurons, consistent with the slow maturation of human neurons.

Thus, perivascular MSCs have a broader stem cell potential than classical neural stem cells. Moreover, perivascular MSCs are not restricted to a certain perivascular niche in neurogenic regions but could be easily isolated from non-neurogenic regions in the brain. Thus, the perivascular MSC is a novel, unique population distinct from the neural stem cells in the adult brain that has both neuroectodermal and mesodermal differentiation capacity *in vitro*. This differentiation capacity was retained in long-term proliferating cultures.

The most intriguing question to be answered now is obviously which role these cells play for disease and repair *in vivo* and whether this reflects their *in vitro* potential.

## Regenerative potential of perivascular mesenchymal stem cells

Perivascular MSCs possess both MSC and pericyte features. Both cell types have been described to have different properties that may play a role in regeneration.

Mesenchymal stem cells *in vitro* have shown several interesting features such as multipotentiality, immunomodulation, and pro-regenerative capacities [9,12,15]. Due to these properties, MSCs have become one of the most promising ASC types and are currently being tested in several clinical trials. Indeed, MSCs are explored as a treatment for Crohn’s disease [67-70], for acute graft versus host reaction [71-73], myocardial infarct [74-76], limb ischemia [77,78], osteogenesis imperfecta [79-81], and for neurological disorders such as stroke [82-84], cerebral palsy [85], amyotrophic lateral sclerosis [86,87] and multiple sclerosis [88-91]. A current search gives a total of 298 clinical studies using different sources of MSCs and mesenchymal stromal cells ( http://www.clinicaltrials.gov). In most of these clinical trials, MSCs are used in an autologous and allogenic *ex vivo* transplantation setting for repair.

Akin to MSCs, pericytes have been reported to be able to differentiate into osteoblasts [25,63,64], chondrocytes, adipocytes [25,65,66], muscle cells [25,92], but also neuroectodermal lineages [32,93].

It remains to be answered whether and to what extent these described *in vitro* properties reflect the *in vivo* function of perivascular MSCs as these properties might be altered upon isolation and culture *in vitro*.

## Multipotentiality *in vivo*

*In vivo* studies are rare due to the ambiguity in markers, but there is some promising evidence that suggest that pericytes may serve as an *in vivo* source of stem or progenitor cells for adult tissue repair [94,95]. Under pathological conditions, a tissue-specific differentiation capacity of pericytes has been observed. Pericytes differentiate into adipocytes during fat tissue injury [29,96], into chondroblasts and bone after bone injury [64] and are the progenitors of Leydig cells of the testis [97]. Genetic lineage tracing reveals that pericytes form odontoblasts during tooth growth and damage *in vivo*[46]. They also contribute to myocytes in skeletal muscle during development and repair [98] and are more frequent in muscles of myopathic patients compared to controls [99]. Furthermore, pericytes are progenitors of follicular dendritic cells in lymphoid follicles [100], they are the origin of myofibroblasts in kidney fibrosis [101], and at least a subtype of pericytes contributes to scar formation in the spinal cord [102]. Resident perivascular MSC give rise to myofibroblasts following lens injury and contribute to fibrogenesis in human lung allografts [103,104] (for summary see Table 2).

**Table 2:** In vivo multipotency of pericytes/perivascular MSC

**Cell origin**	**Markers**	**Experimental model**	**Differentiated cell type**	**Reference**
Vascular progenitor	Nestin	Chemical ablation in testis	Leydig cells	[97]
Pericyte	Alkaline Phospatase	Muscle injury	Myoblast/satellite cell	[98]
Vascular pericyte	Stro-1	Bone injury	Chondroblast/ osteoblast	[64]
Pericyte	NG2	Dental injury	Odontoblast	[46]
Type A pericyte	Glast	Spinal cord injury	Scar tissue/Fibroblast	[102]
Pericyte	PPARγ	Genetic fate mapping	White Adipocyte	[29]
Pericyte	Foxd1 PDGFR-β	Kidney Injury	Myofibroblast	[101]
Perivascular progenitor	PDGFR-β	Genetic fate mapping	Follicular dendritic cell	[100]

## Immunomodulation

Besides their ability to differentiate into cell types from different lineages, isolated MSCs also have an immunomodulatory role [12,21-23].

They have been shown to have an inhibitory effect on lymphocytes [105,106], on B-cells [107], dendritic cells [108] and natural killer cells [109,110]. Furthermore, MSCs modulate the inflammatory response of microglial cells, resident immunocompetent cells in the brain [111]. Mesenchymal stem cells hereby inhibit the expression and release of inflammatory molecules and stress-associated proteins and change microglial cells from a detrimental to a more neuroprotective phenotype [112]. Thus, these immunomodulatory features of MSCs may have an indirect neuroprotective effect [113]. Mesenchymal stem cells lead to amelioration in multiple sclerosis through inhibition of the pathogenic immune response and the release of neuroprotective molecules [22]. They have also been shown to suppress ischemia-induced inflammation [114]. The neuroprotective effect of MSCs in stroke was also mediated via a change in resident microglia to a more neuroprotective type [115]. Furthermore, in a model of Parkinson’s disease, dopaminergic cell death that was induced by activated microglia could be prevented by grafting MSCs [116]. Similar results, demonstrating decreased activation of astrocytes and microglia by MSCs in a mouse model of multiple system atrophy have recently been reported [117,118].

Similarly to MSCs, pericytes have been described to regulate T-cell activation, recruit T- and B-lymphocytes to areas of tissue injury [119,120] and control transmigration of thymocytes from the thymus across the blood vessel wall [121]. In addition, brain pericytes have been shown to secrete different cytokines *in vitro*[122].

Should these immunomodulatory features be present on resident perivascular MSCs *in vivo*, they could indeed play a primary role in inhibiting immunosurveillance and thereby establish a regenerative environment [11].

## Pro-regeneration

A third, and most important feature of isolated MSCs is their pro-regenerative capacity. Mesenchymal stem cells secrete a large number of cytokines, growth factors, mitogens and angiogenic factors [12,95]. This raises the question of whether MSCs could also be promoting a regenerative environment by production of growth factors and cytokines *in vivo*[11].

The most interesting scientific question now is whether their *in vivo* perivascular counterparts hold similar properties mentioned above. It is conceivable that resident perivascular MSCs support the local ASC niche either directly by differentiating into tissue-specific cells as indicated above, or indirectly, by regulating the stem cell niche [123]. Interestingly, pericytes have been shown to contribute to tissue repair and wound healing *in vivo* by substantially enhancing the tissue-regenerative capacity of human epidermal cells [124].

The HSC niche, where MSCs were first identified, is currently the best characterized example of an ASC niche *in vivo* function of resident MSC in the HSC niche was recently revealed by lineage-tracing using nestin as a marker for MSC. This data suggests that resident MSCs are responsible for the maintenance of the HSC niche by regulating the proliferation and survival of HSCs [16].

## Do perivascular mesenchymal stem cells/pericytes play a role in brain repair?

Whether the properties and functions of perivascular MSCs vary between tissues or whether these cells are biologically equivalent will need to be systematically evaluated. The diversity of pericytes is largely unexplored, but there are indications that pericytes in the brain may have specific potential and functions [119,123,125-127]. The brain is one of the most vascularized organs and pericytes have a higher density in the brain, and the brain has a lower endothelial/pericyte ratio compared to other organs [38]. Consistent with their higher density, pericytes appear to act as a key modulator of the neurovascular unit in the brain [123]. Neurovascular pericytes regulate the blood brain barrier [123,126], capillary flow, angiogenesis [128] and immune responses [37,39,41,129,130]. Minor disturbances in the blood vessels can compromise neuronal performance because of the importance of the vasculature for neuronal homeostasis, delivery of oxygen and nutrients, removal of metabolic waste and preservation of the neuronal microenvironment [131]. This is reflected in the fact that vascular damage in pericyte-deficient mice preceeds neuronal damage and neurodegeneration, suggesting that neurodegeneration may develop secondary to disturbances in cerebral vascular homeostasis [127]. Thus, microvascular dysfunction due to pericyte degeneration may initiate neurodegenerative changes [123]. Resident perivascular MSCs may thus regulate the local ASC niche. Another hypothesis could be that pericytes respond to injury by tissue-specific differentiation as evident from other organs (Table 2). Pericytes have been shown to migrate in response to traumatic brain injury [132]. Recent studies that have isolated brain pericytes indicate that the differentiation potential of brain-derived pericytes *in vitro* extends beyond the mesodermal lineage to the neuroectodermal lineage [32,93]. This may at least partially reflect their inherent differentiation potential and could, in analogy to emerging studies in other tissues, possibly indicate their *in vivo* capacity.

However, the role that is played by these cells in brain development and repair remains most speculative and yet, represents one of the most fascinating questions to be raised. It now remains to be shown whether perivascular MSCs/pericytes resident in the brain have similar or equal functional characteristics *in vivo*, supporting the stem cell niche and controlling stem cell proliferation and differentiation. This could place resident perivascular MSCs in a crucial position for contributing to brain disease and regeneration, as much pathology has been associated with a dysregulation of the stem cell niche.

We believe that the properties of these cells observed in other tissues may also apply to the brain. Thus, from a therapeutic perspective, resident MSCs emerge as an extremely promising target or agent for tissue regeneration.

## Conclusion

In a time when the world’s population is aging, the health burden of neurodegenerative diseases such as Alzheimer’s disease and Parkinson’s disease but also conditions such as stroke is constantly increasing. To manage the larger number of patients and the connected health costs, brain research will have to direct a sharp focus towards developing neurorestorative and neuroprotective treatments.

In the next few years, the focus will be on studying the *in vivo* function of the newly discovered perivascular stem cells in the brain. Evidence from *in vitro* work and *in vivo* observations in other tissues gives hope that these perivascular stem cells may play a key role for regeneration of the brain in response to trauma, injury or degeneration. The aim is to control and enhance any pro-regenerative capacities of these cells by delivering therapies targeted at stimulating the cells to relocate to sites of injury or damage.

To understand and harness the reparative potential of ASCs in the brain will be key in setting the course for future research on neurodegeneration and neurorestoration.

## Abbreviations

ASCs: Adult Stem Cells; MSCs: Mesenchymal Stem Cells; HSCs: Hematopoietic Stem Cells; CD105: Endoglin cell membrane glycoprotein; CD13: Cluster differentiation marker; PDGFRβ: Platelet-Derived Growth Factor Receptor beta; FACS: Fluorescence-Activated Cell Sorting; CD45: Leukocyte common antigen; CD31: Pan-endothelial marker; CD73: Ecto-5′-nucleotidase (NT5E). GPI-anchored purine enzyme expressed on the surface of human T and B lymphocytes; CD90: Thymocyte differentiation antigen 1 (Thy-1); CD106: Vascular cell adhesion protein 1 (VCAM-1); CD49d: Alpha subunit of integrin alpha4beta1; RGS5: Regulator of G-protein Signaling 5; α-SMA: Alpha Smooth Muscle Actin; NG2: Chondroitin Sulfate Proteoglycan; NGN2: Neurogenin 2; CD133: Prominin 1; SOX1: Sox gene 1; PAX6: Paired box protein 6; GFAP: Glial Fibrillary Acidic Protein; Tbr1: T-box brain 1 transcription factor; Tbr2: T-box brain 2 transcription factor; DCX: Doublecortin; Tuj1: Neuronal class III beta-tubulin; HuC/D: Anti human neuronal protein HuC/D; Map2: Microtubule-associated protein 2; NSE: Neuron-specific Enolase; PSD95: Postsynaptic Density Protein 95; GABA: Gamma-Aminobutyric Acid; Kir6.1: Potassium channel subunit; A2B5: Ganglioside marker; S100b: S100 calcium-binding protein; GLAST: Astrocyte-specific transporter; O4: Oligodendrocyte marker; CD34: Single chain transmembrane glycoprotein selectively expressed on human lymphoid and myeloid hematopoietic progenitors cells; CD166: Activated leukocyte cell adhesion molecule; CD56: Neural cell adhesion molecule (N-CAM); CD14: Cluster of differentiation 14, cell surface receptor and differentiation marker; CD11b: Macrophage antigen 1 (Mac-1); Stro1: Mesenchymal/stromal stem cell marker 1; PPARγ: Peroxisome proliferator-activated receptor gamma; Foxd1: Forkhead box transcription factor 1.

## Competing interests

The authors declare that they have no competing interests.

## Authors’ contributions

IÖ, JB and GP wrote the manuscript. GP made the design for Figure 1. IÖ contributed the tables and figure legends. All authors read and approved the final manuscript.
